# Cost analysis and cost-effectiveness of traditional Chinese medicine in lung cancer: a scoping review

**DOI:** 10.3389/fpubh.2025.1669751

**Published:** 2025-11-03

**Authors:** Ran An, Gangqiang Su

**Affiliations:** ^1^Dongzhimen Hospital, Beijing University of Chinese Medicine, Beijing, China; ^2^Nanjing University of Chinese Medicine, Nanjing, China

**Keywords:** economic evaluation, lung cancer, traditional Chinese medicine, cost, cost-effectiveness analysis

## Abstract

**Background:**

Lung cancer remains the leading cause of cancer mortality worldwide, with non-small cell lung cancer (NSCLC) comprising the majority of cases. Traditional Chinese Medicine (TCM) has been increasingly integrated into lung cancer care, particularly in East Asia, but its economic impact remains unclear.

**Methods:**

A scoping review was conducted to synthesize evidence on the cost and cost-effectiveness of TCM for lung cancer. PubMed, Web of Science, and Scopus were searched from inception to July 2025. Eligible studies included economic evaluations comparing TCM with non-TCM treatments or conventional therapies. Outcomes included direct costs, incremental cost-effectiveness ratios (ICERs), and quality-adjusted life years (QALYs).

**Results:**

Eight studies met the inclusion criteria, including cohort studies, cross-sectional analyses, and one randomized controlled trial. The included studies spanned from 2003 to 2024, with data primarily from Taiwan and China. Findings were mixed: some studies reported higher total costs for TCM users due to additive, rather than substitutive use alongside conventional therapies. However, several studies demonstrated favorable ICERs for TCM, especially when administered over longer durations. One study showed an ICER of NT$880,908 per life-year gained, well below Taiwan’s willingness-to-pay threshold. Outpatient TCM use was often more cost-effective than inpatient care, and herbal medicine appeared more economical than patent medicine.

**Conclusion:**

Adjunctive TCM may be cost-effective in lung cancer treatment when integrated thoughtfully within national healthcare systems. Cost outcomes vary by care setting, modality, and region. Future standardized, prospective evaluations are warranted to guide the efficient integration of TCM in oncology.

## 1 Introduction

Lung cancer continues to be the leading cause of cancer-related mortality globally, with approximately 2.2 million new cases and 1.8 million deaths documented in 2020, accounting for 18% of all cancer deaths worldwide (1, 2). Non-small cell lung cancer (NSCLC) accounts for over 85% of all lung cancer cases and is often identified at advanced stages, resulting in a poor prognosis and five-year survival rates that mostly fall below 20% (3, 4). Lung cancer is a major public health issue in China, ranking as the leading cause of cancer death and incidence for men and second for women, which imposes a substantial burden on patients and the health care system (5).

With thousands of years of background, Traditional Chinese Medicine (TCM) has been considered a complementary therapy for various types of cancer in China and other East Asian healthcare systems in recent decades. TCM includes, but is not limited to, herbal remedies, acupuncture, moxibustion, qigong, and dietary modifications (6). Its use is profoundly embedded in Chinese cultural and medical traditions (7, 8) and has been progressively incorporated into national healthcare systems, including Taiwan’s National Health Insurance and China’s Urban Basic Medical Insurance programs (9, 10). A study conducted across fourteen European countries found that 23.6% of lung cancer patients used complementary and alternative medicine. This rate was slightly higher than that of head and neck cancer patients (22.7%) but significantly lower than the 56.3% reported among pancreatic cancer patients (11). A study on the determinants of TCM utilization of patients with cervical, breast, lung, liver, or colorectal cancers in Taiwan has shown that the prevalence of TCM use for lung cancer patients was 16.03% (12).

Existing evidence suggests that TCM can be applied as an adjunct in the treatment of lung cancer in improving patients’ quality of life, enhancing the efficacy of conventional treatments synergistically, and extending survival. For instance, adjuvant TCM treatment reduced mortality for stage IV patients significantly when applied for 6 months or more in a Taiwan nationwide cohort study of NSCLC patients (13). A systematic review, which analyzed 24 clinical trials, assessed the effectiveness of combining Chinese herbal medicine with conventional chemotherapy in treating advanced NSCLC. The review identified the five most frequently used herbs as *Radix Adenophorae*, *Radix Ophiopogonis*, *Radix Glycyrrhizae*, and *Poria.* Adjunct herbal medicine may help reduce chemotherapy-related side effects such as nausea, vomiting, reductions in hemoglobin levels, white blood cell suppression, and platelet decline. Additionally, it appeared to improve the one-year survival rate, boost short-term tumor response, and enhance the Karnofsky performance score in patients with advanced NSCLC (14). Another systematic review involving 862 patients with NSCLC found that oral Chinese herbal medicine, when combined with chemotherapy, can enhance patients’ quality of life, tumor response, and survival rate, while also alleviating cancer-related symptoms (15). In addition, a randomized controlled trial showed that stage-specific TCM combined with chemotherapy significantly improved both survival and quality of life in patients with advanced NSCLC (16).

Further evidence supports TCM’s potential survival benefit. TCM reduced mortality at most by 32% in longitudinal analysis (17). In addition, Li et al. identified specific herbal formulations, i.e., Bu-Zhong-Yi-Qi-Tang, Xiang-Sha-Liu-Jun-Zi-Tang and Bai-He-Gu-Jin-Tang, as being associated with significantly improved overall outcomes (18, 19). At a biological level, TCM medications have been found to trigger apoptosis, reduce metastasis, reverse drug resistance, and reduce chemotherapeutic toxicity in preclinical and translational studies (20, 21).

Despite these encouraging results regarding the effectiveness of TCM in lung cancer treatment, the financial effects of using TCM to treat lung cancer are still unknown. Although certain studies indicate possible cost-effectiveness, others have documented markedly elevated healthcare costs among TCM users. For example, comprehensive national research in China revealed that TCM users experienced greater inpatient expenses compared to non-users (22). These conflicting results underscore the need for a comprehensive evaluation of the financial implications and cost-effectiveness of TCM in lung cancer treatment.

In this scoping review, we aimed to consolidate existing evidence regarding the costs associated with TCM utilization in lung cancer patients, emphasizing both total expenses and cost-effectiveness. Comprehending the financial implications of TCM is essential for doctors, patients, and policymakers involved in integrative oncology care models in high-burden environments.

## 2 Methods

### 2.1 Study design

This scoping review aims to describe, summarize, and facilitate the dissemination of research findings regarding the economic evaluations, including cost analysis and cost-effectiveness analysis of TCM in lung cancer. This review provides a narrative and descriptive explanation of available research, considering the methodological framework described previously (23). For this scoping review, we applied the following definition for TCM: TCM is a comprehensive system of medical theory and practice that integrates various therapeutic modalities, including Chinese patent medicine, Chinese herbal medicine, acupuncture, moxibustion, cupping therapy, and Qigong (breathing and movement exercises). However, it should be noted that although TCM includes various modalities, the most extensively studied and widely accepted components in the treatment of lung cancer are Chinese herbal medicine and Chinese patent medicine. Based on our preliminary search, these two pharmacologic approaches are the primary forms of TCM evaluated in cost-effectiveness research, while other modalities are generally applied for symptom management or quality-of-life enhancement rather than direct oncologic treatment. Therefore, we primarily considered pharmacologic modalities of TCM, specifically Chinese herbal medicine and Chinese patent medicine, as the focus of economic evaluation. Non-pharmacologic treatments were included only when clearly evaluated in cost-related outcomes.

### 2.2 Eligibility criteria

We included original research studies that evaluated the cost, economic burden, or cost-effectiveness of TCM interventions in patients with lung cancer, irrespective of cancer subtype or stage. Eligible studies included randomized controlled trials (RCTs), retrospective or prospective cohort studies, cross-sectional studies, and economic evaluations. The primary outcomes of interest were direct medical costs, incremental cost-effectiveness ratios (ICERs), cost-utility analyses, and comparative inpatient/outpatient healthcare expenditures. Studies were included if they compared TCM use to non-TCM treatment or to standard Western medicine (WM) alone. We also included studies which only included TCM users and reported cost analysis in a specific context. Both English and Chinese-language publications were eligible. Reviews, commentaries, editorials, case reports, and studies without original cost data were excluded.

### 2.3 Data sources and search strategy

We performed a comprehensive literature search in three electronic databases: PubMed, Web of Science, Scopus, CNKI, and Wanfang from inception through July 2025. We also updated our search in September 2025. The search strategy combined keywords related to lung cancer, TCM, and economic evaluation using Boolean operators, and was applied across five databases. Search keywords and their alternatives are represented in Supplementary Table S1. Supplementary Table S2 shows the detailed search strategy and results in each database. This search was further supplemented by a manual search via backward citation searching of relevant studies and Google Scholar to ensure not missing relevant studies.

### 2.4 Study selection

After removing duplicates using the automatic “Find Duplicates” feature in EndNote (X8.0.2), two reviewers independently screened titles and abstracts for potential inclusion, followed by full-text reviews. Discrepancies were resolved through discussion or adjudication by a third reviewer. The final selection was documented in a PRISMA flow diagram.

### 2.5 Data extraction

A standardized data extraction form was used to collect relevant information, including study characteristics (First author, publication year, country, study design, data source), population (sample size, cancer stage, diagnosis code system), type of TCM modality, care setting, groups, cost outcomes (total costs, ICER, cost-effectiveness outcomes), and key findings.

### 2.6 Cost standardization

To enable meaningful comparison across studies, all cost values were standardized to 2023 US dollars (USD) using a two-step process. First, reported costs were inflated to 2023 values using country-specific Consumer Price Index (CPI) data. Second, inflated values were converted to USD using Purchasing Power Parity (PPP) conversion rates sourced from the World Bank. For Chinese studies, a PPP factor of 1.35 RMB/USD was applied, while for Taiwanese studies, 13.883 NT$/USD was used. For studies that did not report the price year, the publication year was used as a proxy.

### 2.7 Data synthesis

Due to heterogeneity in economic outcome definitions and methodologies, we performed a narrative synthesis. Key cost metrics and ICERs were tabulated and summarized, if available. Statistical significance values were extracted and reported when available in the included studies. No meta-analysis was planned due to expected methodological variation across included studies.

### 2.8 Quality assessment

A quality appraisal was conducted for all included studies using standardized tools from the Joanna Briggs Institute (JBI). The JBI critical appraisal checklists for randomized controlled trials, cohort studies, and cross-sectional studies were applied based on study design (24–26). Each study was independently assessed by two reviewers, and discrepancies were resolved through consensus.

## 3 Results

**Figure 1:**
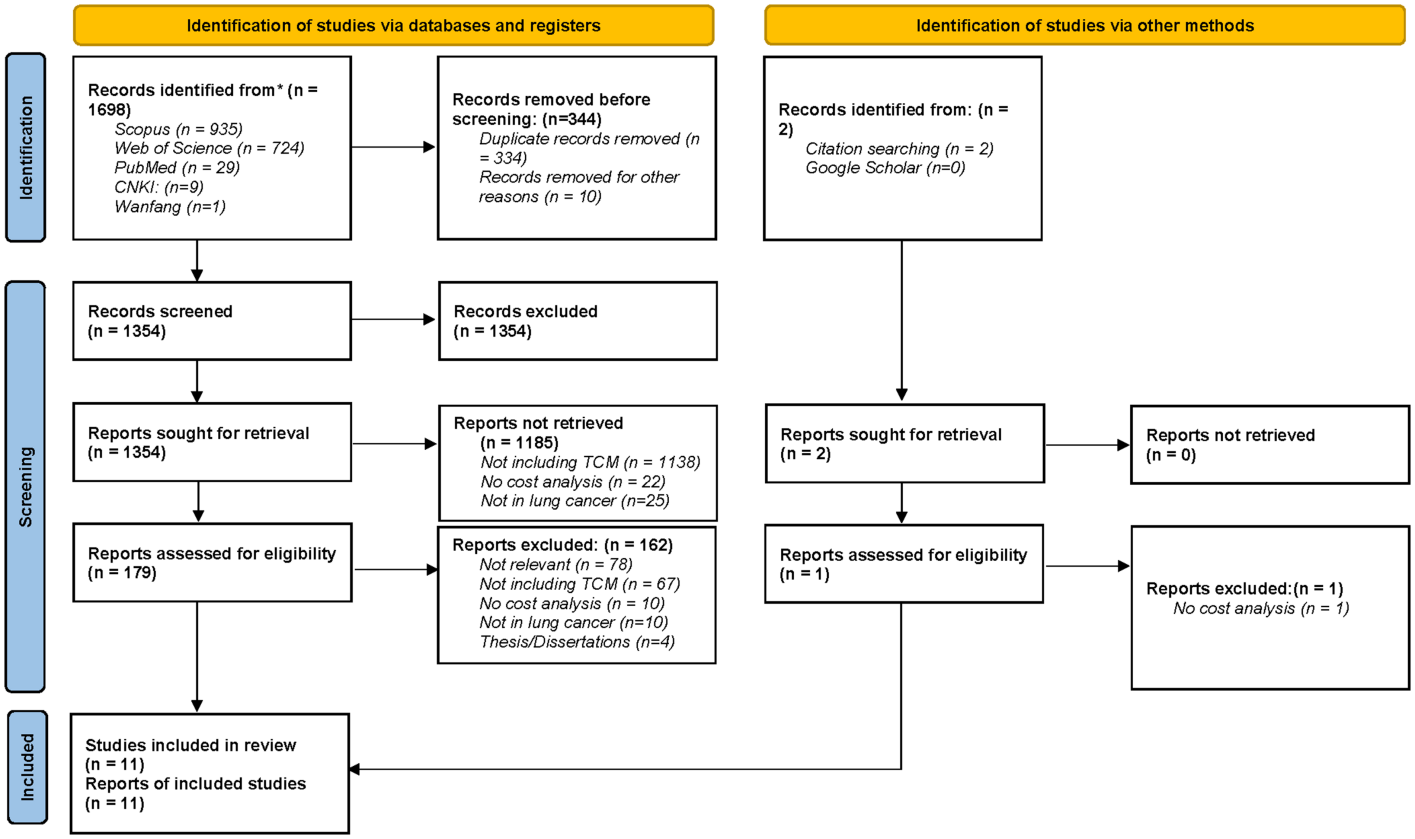
PRISMA 2020 flow diagram for this scoping review which included searches of databases, registers and other sources.

A total of 1,698 records were identified through database searches and manual reference screening. After removal of duplicates and ineligible publications (*n* = 344), 1,354 titles and abstracts were screened. Of these, 179 full-text articles were assessed for eligibility. Ultimately, 11 studies (13, 27–36) (five studies in English and six studies in Chinese) met the inclusion criteria and were included in the final synthesis (Figure 1).

### 3.1 Study characteristics

The included studies were published between 2003 and 2024, and most of them used real-world data from large national or regional health insurance databases in Taiwan and China.

A total of 11 studies reported the cost analysis or cost-effectiveness of TCM use in patients diagnosed with lung cancer, as shown in Table 1. The included studies comprised seven retrospective cohort studies (13, 28, 32, 33), three cross-sectional studies (29–31), and one randomized clinical trial (27), reflecting diverse methodological approaches to evaluating the economic impact of TCM in lung cancer.

**Table 1:** Study characteristics and cost measures of included studies.

References	Design, data source (years)	Population, cancer stages diagnosis code system	TCM modality, care setting	Groups	Cost measures	Standardized costs (2023 USD, PPP-adjusted)	Cost difference/ICER	Key findings
Tang et al. (13) (Taiwan)	Retrospective cohort, NHIRD (2000–2018), Cancer Registry, Cause of Death Data, Household Registration Database (2004–2014) [final inclusion 2007–2013]	13,484 NSCLC patients, I-IV, ICD-O-3 (codes: C33 ~ C34)	Adjunctive Chinese medicine, NA	TCM users/ non-TCM users: 2,308/11,540	Average total cost in TCM users vs. non-users: NT$1,385,021 vs. NT$1,236,988 over 5 years	Average total cost in TCM users vs. non-users over 5 years: $97,743 vs. $87,306	ICER ≈ NT$880,908 per life-year gained	The cost-effectiveness of patients receiving adjunctive TCM therapy falls within an acceptable range (within 3 times GDP) for primary diagnosis costs, primary and secondary diagnosis costs, and total costs.
Nie et al. (30) (China)	Cross-sectional study, CHIRA (2010–2016)	47,393 lung cancer patients, NA, ICD-10 (NA)	Chinese herbal medicine, Chinese patent medicine, and Chinese medicine injection, inpatients	TCM users/ non-TCM users: 8,696/38,715	Median total medical cost in TCM users vs. non-users: RMB18,798 (USD2,830) vs. RMB8,001 (USD1,205) [*p* < 0.001]	Median total medical cost in TCM users vs. non-users: $13,925 vs. $5,927	65.2% higher cost of TCM users vs. non-users	TCM users incur higher inpatient costs. The medication cost, conventional medication cost, and nonpharmacy cost of TCM users were all higher than TCM nonusers, illustrating the higher medical cost of TCM users was not induced by TCM only.
Liao et al. (28) (Taiwan)	Retrospective cohort study, LHID (1996–2010)	7,677 lung cancer patients, NA, ICD-9-CM (code: 162)	TCM services, ambulatory or inpatient	Non-surgery (TCM users-non-TCM users)/ surgery (TCM users-non-TCM users): 6939 (5113–1826)/738 (508/230)	Total cost of non-surgery vs. surgery groups in TCM users vs. TCM non-users: NT$55,620,684 vs. NT$167,168,555 and NT$8,426,264 vs. NT$16,282,213	Total cost of non-surgery vs. surgery groups in TCM users vs. TCM non-users: $4,792,982 vs. $14,406,759 and $726,095 vs. $1,403,492	Lower cost of TCM users vs. non-users in the outpatient context	The total amount paid per visit for WM is higher than that for 1 year of TCM outpatient care before and after lung cancer diagnosis.
Liu et al. (33) (China)	Retrospective cohort study, 47 hospitals in Beijing (2019)	12,564 lung cancer patients, NA, NA	Chinese herbal medicine, Chinese patent medicine, inpatient	NA	NA	NA	NA	The cost of inpatients with lung cancer was higher when Chinese patent medicine was involved, and lower when Chinese herbal medicine was involved. The participation rate of Chinese patent medicine in patients with lung cancer is about 50%, and the participation rate of Chinese herbal medicine is relatively low.
Liu et al. (29) (China)	Cross-sectional study, 6 hospitals in Guangzhou (2007)	608 NSCLC patients, IIIB-IV, NA	TCM services, inpatient	TCM users/integrated TCM and WM/ WM: 106/343/159	NA	NA	62.95 and 28.51% lower cost of TCM and integrated TCM and WM vs. WM only services	The inpatient costs of TCM and integrated TCM and WM for the treatment were lower than the cost of WM.
Zhu et al. (31) (China)	Cross-sectional study, multiple hospitals in China (NA)	326 NSCLC patients, IIIB-IV, NA	Kang Ai injection and Shenqi Fuzheng, inpatient	Combination of Kang Ai injection and chemotherapy vs. combination of Shenqi Fuzheng and chemotherapy: NA	Total cost of Kang Ai injection group vs. Shenqi Fuzheng injection group: USD5,997.32 vs. USD5,893.94	Total cost of Kang Ai injection group vs. Shenqi Fuzheng injection group: $5,937.35 vs. $5,835.00	ICER derived from Kang Ai injection group compared with Shenqi Fuzheng injection group≈ $476.41/QALY	Kang Ai injection combined with chemotherapy appeared to be more cost-effective than Shenqi Fuzheng injection combined with chemotherapy. ICER derived from Kang Ai injection group compared with Shenqi Fuzheng injection group was less than the willingness to pay threshold of one time the GDP per capita.
Wu et al. (32) (China)	Nationwide Cohort study, URBMI (2008–2010)	51,382 cancer patients (the number of Lung cancer patients: NA), NA, NA	Chinese patent medicines, inpatient	TCM vs. WM: NA	Total cost of TCM vs. WM: RMB2470.59 vs. RMB3831.93	Total cost of TCM vs. WM: $1,948.56 vs. $3,025.70	Higher cost of WM vs. TCM	The TCM was most often used in lung cancer patients (52%). The total cost for WM is higher than that for TCM in the inpatient setting.
Bai et al. (27) (China)	Randomized crossover trial, hospital in Shaanxi Province (NA)	123 lung cancer patients, II-IV, NA	Shenqi Fuzheng injection, inpatient	Combination of Shenqi Fuzheng injection and chemotherapy vs. chemotherapy	NA	NA	220.5 more Chinese yuan for the combination group vs. the chemotherapy group	The chemotherapy cycles with Shenqi Fuzheng injection cost more, but the adverse effects were slightly different, and the change of QOL domains showed significantly better results as compared to the chemotherapy group.
Zhao et al. (34) (China)	Retrospective cohort, hospital-based; PLA General Hospital (2013–2015)	400 NSCLC inpatients, stage III–IV, Diagnosis based on imaging and pathology, no ICD code reported	TCM injections and decoctions, inpatient	Chemo only vs. Chemo + TCM injection vs. Chemo + TCM individualized (syndrome differentiation)	Direct medical costs (hospital billing)	NA	Chemo + TCM individualized (syndrome differentiation): lowest cost per effective case (¥24,899.49) and the highest effectiveness (90%)	TCM individualized group had higher clinical effectiveness and lower cost per effective case than WM alone.
He et al. (35) (China)	Retrospective cohort, hospital-based; Guangzhou Chinese Medicine Hospital (2006–2008)	200 NSCLC inpatients, NA, NA	Chinese herbal medicine (oral), inpatient	TCM only vs. WM only vs. Integrative (TCM + WM)	Direct medical costs (per inpatient episode)	NA	ICER for Integrative vs. WM alone = ¥44.97 per unit of effectiveness gain with the highest effectiveness (91.5), with a moderate cost increase over WM alone.	Integrative group showed better symptom relief and cost-effectiveness compared to WM alone.
Chang-ming et al. (36) (China)	Retrospective cohort, hospital-based; Shanxi Provincial Hospital (2014–2017)	270 NSCLC patients, stage III–IV, Diagnosis based on clinical and pathological criteria	TCM herbal decoction + injections, inpatient	WM vs. Integrative (chemo + TCM)	Direct inpatient medical costs	NA	CER for WM: ¥23,710.42; Integrative (chemo + TCM) group B: ¥18,137.56 per effective case.	Integrative TCM group showed higher effectiveness (76.6%) at a lower cost per success, indicating better cost-effectiveness.

LHID: Longitudinal Health Insurance Database; WM: Western medicine; ICD: International Classification of Diseases; CM: Clinical Modification; ICD-O-3: International Classification of Diseases for Oncology, third edition; CHIRA: China Health Insurance Research Association; NHIRD: National Health Insurance Research Databases; ICER: Incremental cost-effectiveness ratio; RMB: Renminbi; USD: United States Dollar; NT$: New Taiwan Dollar; URBMI: urban basic medical insurance.

Two were large retrospective cohort studies using claims data from Taiwan’s National Health Insurance Research Database (NHIRD), and one was a cross-sectional study using a 5% random sample of inpatient claims from the China Health Insurance Research Association (CHIRA). All studies evaluated the economic impact or cost-effectiveness of TCM use in patients diagnosed with non-small cell lung cancer (NSCLC). The characteristics of the included studies are summarized in Table 1.

In the majority of clinical practice settings and studies included in this review, TCM was employed as an adjunctive therapy to conventional Western treatments, rather than as part of a fully integrated therapeutic model.

### 3.2 Data sources

Four of the included studies utilized large-scale, population-based health insurance databases from China and Taiwan (13, 28, 30, 32). Nie et al. used data from the China Health Insurance Research Association (CHIRA), which covers over 93% of the country’s urban residents under the Urban Employee Basic Medical Insurance (UEBMI) and Urban Resident Basic Medical Insurance (URBMI) schemes. They included lung cancer inpatients from 2010 to 2016, with a 5% random sample drawn using systematic sampling principles (30). Similarly, Wu et al. utilized this data source, covering data from 2008 to 2010 (32). In Taiwan, researchers accessed the National Health Insurance Research Database (NHIRD), a comprehensive dataset representing about 99% of the Taiwanese population. Several datasets were utilized, including the Longitudinal Health Insurance Database 2005 (LHID2005), which contains detailed registration data for a random sample of one million beneficiaries between 1996 and 2010, and broader NHIRD data spanning 2000–2018, as well as complementary datasets from the Cancer Registry, Cause of Death Database, and Household Registration Database (13, 28). Among the remaining studies that included more than 1,000 patients in their assessment, Liu et al. collected sociodemographic and clinical data of hospitalized patients with lung malignancy from 47 hospitals in Beijing, covering the period from January 1, 2019, to December 31, 2019. This dataset provided detailed insight into real-world hospital-based characteristics of lung cancer patients in a major urban center (33).

### 3.3 Population

Overall, 83,045 patients with lung cancer were included in this study. The highest sample size was for Tang et al. study who identified 76,232 newly diagnosed non-small cell lung cancer (NSCLC) patients from Taiwan’s Cancer Registry (2007–2013). After applying exclusion criteria related to unclear diagnosis, small cell lung cancer, prior malignancies, or lack of treatment, they finally excluded 43,122 patients. A final cohort of 13,848 patients (2,308 TCM users and 11,540 non-users) was included using 1:5 propensity score matching to reduce bias in this study (13).

### 3.4 Care setting

Based on available evidence, only one study assessed both outpatient and inpatient costs of TCM (28), and all other studies assessed the cost and cost-effectiveness in an inpatient setting (13, 27, 29–33).

### 3.5 Cost outcomes

#### 3.5.1 General cost analysis

Nie et al. (2023) analyzed inpatient costs of 47,393 lung cancer patients in China, reporting significantly higher total median hospitalization costs for TCM users (RMB 18,798 or USD 2,830) compared to non-users (RMB 8,001 or USD 1,205; *p* < 0.001). The higher costs were observed across all age groups, hospital levels, and regions, and were attributed not only to TCM expenses but also to increased conventional medication and non-pharmacy costs, indicating additive rather than substitutive use of TCM (30). Similarly, Tang et al. reported that NSCLC patients in Taiwan receiving adjunctive TCM therapy incurred higher average total costs over 5 years compared to non-TCM users—NT$1,385,021 vs. NT$1,236,988. However, when evaluated in terms of cost-effectiveness, TCM users demonstrated an ICER of NT$880,908 per life-year gained, falling within the acceptable threshold of three times GDP per capita in Taiwan (13). In contrast, Wu et al. found that in a nationwide cohort study using China’s urban basic medical insurance data, inpatient Western medicine (WM) costs exceeded those of TCM (RMB3,831.93 vs. RMB2,470.59) (32). In another study, Liu et al. examined economic outcomes across three treatment groups in six hospitals in Guangzhou and showed that both TCM and integrated TCM-Western medicine (WM) groups had significantly lower inpatient costs than WM alone (29). After standardizing all cost estimates to 2023 PPP-adjusted USD, the variation in total cost across studies remained substantial, though more interpretable. For example, in Tang et al. study, costs for TCM users were $97,743 vs. $87,306 for non-users, while in Wu et al. study, the cost was $1,949 for TCM vs. $3,026 for WM. These differences reflect both clinical variability and differences in healthcare delivery models.

#### 3.5.2 Cost-effectiveness analysis

Only three studies performed a cost-effectiveness analysis for TCM therapy in lung cancer patients (13, 27, 31). Tang et al. showed that the presence or absence of adjunctive TCM therapy was not significantly associated with patient survival or death. However, analysis based on the duration of TCM use demonstrated a significant difference in patient survival and death, especially among those using TCM for 181–365 days, effectively reducing the mortality rate to 65.10%. Patients who received adjunctive TCM therapy for 181–365 days showed a significantly lower risk of mortality (HR = 0.88, 95% CI: 0.80–0.98) and a mean increase in survival of 0.17 life-years over 5 years. While the costs for patients with adjunctive TCM therapy were higher than those without adjunctive TCM therapy (annual total costs per person for those without adjunctive TCM therapy: NT$ 1,236,988, vs. for those with adjunctive TCM therapy: NT$ 1,385,021.), the incremental cost-effectiveness ratio (ICER) was estimated at NT$880,908 per life-year gained, well below Taiwan’s willingness-to-pay threshold of 1–3 × per capita GDP (NT$1.2–NT$3.6 million), indicating high cost-effectiveness (13). In another cost-effectiveness analysis, Zhu et al. found that Kang Ai injection, when combined with chemotherapy, provided an incremental gain of 0.217 QALYs at an additional cost of $103.38 compared to Shenqi Fuzheng injection, yielding an ICER of $476.41/QALY. This figure is well below China’s willingness-to-pay threshold of $12,070/QALY (2022 GDP per capita). Sensitivity and scenario analyses confirmed the robustness of these findings, even when the time horizon was shortened to 5 years (ICER: $4,081.83/QALY) (31). In another study, Zhao et al. demonstrated that integrative TCM, particularly individualized decoctions based on syndrome differentiation, offered the highest clinical effectiveness (90%) at the lowest cost per effective case (¥24,899.49) compared to WM or generic TCM injections (34). He et al. calculated an ICER of ¥44.97 per unit of effectiveness gain when comparing integrative therapy to WM alone, indicating favorable cost-effectiveness (36). Similarly, Chang-ming et al. reported a cost-effectiveness ratio (CER) of ¥18,137.56 per effective case in the integrative group versus ¥23,710.42 in the WM group, suggesting better economic value for integrative care in advanced NSCLC inpatients (35). Moreover, Bai et al.’s randomized crossover trial showed that the addition of Shenqi Fuzheng injection to chemotherapy modestly increased treatment costs but yielded notably improved quality-of-life outcomes across multiple domains, highlighting the potential for enhanced value despite higher direct costs (27).

#### 3.5.3 Cost variability by TCM modality

Liu et al. observed mixed cost outcomes depending on the TCM modality. The use of Chinese patent medicine in hospitalized lung cancer patients was associated with higher overall costs, whereas the use of Chinese herbal medicine correlated with lower costs (33). Liao et al. supported this cost-saving effect, noting that the average amount paid per WM visit exceeded the annual cost of TCM outpatient care both before and after lung cancer diagnosis (17). Further evidence for the economic benefits of TCM comes from Liu et al., who demonstrated that inpatient treatment costs for stage IIIB–IV NSCLC were 62.95 and 28.51% lower for patients receiving TCM alone or integrated TCM-WM therapy, respectively, compared to WM alone (29).

#### 3.5.4 Outpatient TCM costs

As noted above, Liao et al. evaluated outpatient visits in lung cancer patients in Taiwan and found that while TCM users had more frequent visits, the annual outpatient cost for TCM was lower than that for WM. This suggests potential for cost containment in outpatient settings, despite increased healthcare utilization (28).

#### 3.5.5 Quality assessment

The results of the quality appraisal are provided in Supplementary Table S3. The overall quality was moderate to high across most studies. Most cohort studies clearly defined their populations and outcomes, and addressed confounding through matching or statistical adjustment. However, several studies had limitations regarding the reporting of follow-up completeness and outcome assessor blinding, particularly in retrospective designs. The RCT by Bai et al. met most methodological criteria. Cross-sectional studies generally met core quality criteria, though some lacked detailed reporting on measurement validity and confounding control. These findings underscore the heterogeneity in study designs and reporting quality, which should be considered when interpreting the review’s conclusions.

## 4 Discussion

This scoping review summarizes the most recent data from eight studies that report on the cost-effectiveness and economic evaluation of TCM in the treatment of lung cancer. The results show a complex and diverse landscape; although TCM may have favorable cost-effectiveness profiles when used in conjunction with conventional treatments, it is linked to both higher and lower direct costs depending on the context.

One of the key findings across studies is the heterogeneity in cost outcomes associated with TCM use. According to Nie et al., TCM users had 65.2% greater inpatient expenses than non-users, with higher expenditures in the categories of conventional care, non-pharmacy, and medication. This suggests that TCM might be applied as a supplement rather than a replacement treatment, which would result in a higher use of resources (30). Similarly, over a five-year period, Tang et al. discovered that TCM users had higher overall costs (13). On the other hand, Wu et al. and Liu et al. found that patients receiving TCM or integrated TCM-WM therapy had lower costs than those receiving WM alone (29, 32). Important variabilities in study designs, patient populations, healthcare settings (e.g., inpatient vs. outpatient), and TCM modalities (e.g., Chinese patent medicine vs. herbal therapies) are reflected in these contradictory findings. Such discrepancies may also be partly explained by differences in TCM delivery models across regions. For example, Liao et al. highlighted that while TCM users in Taiwan engaged in more frequent outpatient visits, their annual outpatient costs remained lower than those for WM, suggesting a potential role for TCM in cost containment within ambulatory care (17). In contrast, Nie et al.’s data from large Chinese cohorts suggest that inpatient TCM use may increase hospital expenses in urban settings (30). These findings showed that outpatient TCM use may be more economically favorable than inpatient use. Furthermore, these contrasting outcomes highlight a key consideration in evaluating the economic implications of TCM. In general, the health system context and reimbursement structure matter. For instance, in Taiwan, where TCM is fully reimbursed and tightly integrated within the National Health Insurance system, usage patterns may be more standardized and cost-effective. In China, the absence of unified clinical guidelines for TCM and variability in provider practices may lead to inefficiencies or overutilization, thereby inflating costs.

Despite the variability in direct costs, several studies reported favorable cost-effectiveness outcomes for TCM. In Tang et al., adjunctive TCM therapy resulted in a modest increase in survival (0.17 life-years over 5 years), with an ICER of NT$880,908 per life-year gained, well below three times the GDP as the threshold. These findings support the economic value of integrating TCM into standard cancer care, especially when maintained over longer periods.

This finding was further supported by Zhu et al. using data from a cost-utility study of two injectable TCM formulations. When combined with chemotherapy, the Kang Ai injection produced 0.217 QALYs at an additional cost of $103.38, yielding an ICER of $476.41/QALY, which is significantly less than China’s per capita GDP threshold. This finding’s robustness across modeling assumptions was validated by sensitivity and scenario analyses, offering a strong case for its cost-effectiveness (31). By showing better quality-of-life (QOL) outcomes in patients treated with Shenqi Fuzheng injection, albeit with a modest cost increase, Bai et al. contributed qualitative value to this discussion. The results imply that, in certain patient populations, value-based improvements in QOL might justify higher direct treatment costs, even in the absence of a formal cost-utility analysis (27). Notably, recent hospital-based studies from mainland China provide further support for the cost-effectiveness of integrative TCM. Zhao et al. and Chang-ming et al. both demonstrated that individualized or combined TCM interventions led to either superior clinical outcomes or lower cost per success. These findings reinforce the economic viability of personalized, stage-specific TCM applications, particularly in inpatient settings for advanced-stage NSCLC (34, 35).

Differentiating between TCM modalities is crucial, according to available evidence. Liu et al. demonstrated that while Chinese herbal medicines were associated with lower costs, Chinese patent medicines were linked to higher inpatient costs. This distinction is important for both health system budgeting and clinical decision-making. Economic sustainability in the treatment of lung cancer may be improved by customizing TCM use according to the cost-effectiveness of particular modalities.

Furthermore, the duration and integration level of TCM use also influenced outcomes. In Tang et al., TCM use was not significantly associated with survival when analyzed as a binary exposure (user vs. non-user), but showed a dose–response effect, patients using TCM for 181–365 days experienced significantly reduced mortality (HR = 0.88) (13). This finding indicates that longer-term, consistent use of TCM may yield more pronounced benefits, both clinically and economically. Moreover, Future integration of TCM with nanomedicine offers promising strategies to enhance therapeutic efficacy and bioavailability (37).

To better understand the variability in reported cost and cost-effectiveness outcomes, we examined five key study-level factors across the included studies, including cancer stage, TCM modality type, treatment duration, healthcare setting, and integration model. In total, studies including advanced-stage NSCLC or SCLC generally reported greater cost differences between TCM-integrated and non-TCM groups, likely due to prolonged survival and reduced complications. Herbal medicine (decoctions or patent formulations) was the most commonly studied intervention. Studies examining multi-modality approaches reported higher costs but occasionally better outcomes. Moreover, longer TCM use (>6 months or full course) was associated with higher total costs, but several studies indicated lower cost per effective case or improved survival/QALY. In addition, inpatient vs. outpatient use influenced cost substantially. In hospital-based TCM use, costs were consistently higher, but effectiveness outcomes were also more favorable. When TCM was used as adjunctive therapy, it generally led to moderate cost increases with potential improvements in quality-adjusted outcomes. Fully integrative pathways were more cost-effective in several retrospective cohort studies.

Although our scoping review primarily includes studies from Taiwan and mainland China, we explored the existence of any potential evidence from nearby East Asian healthcare systems such as Japan, South Korea, and Singapore. While Kampo medicine is used adjunctively in Japanese oncology for supportive care (38–40), and preclinical studies investigating ginsenosides (e.g., Rh2, Compound K) in Korean research have demonstrated promising anticancer activity (41, 42), we found no published cost-effectiveness or economic evaluation studies of these interventions in the context of lung cancer. This highlights an important gap in the literature and underscores the need for formal health economic assessments in these regions to better understand the generalizability of TCM-related cost-effectiveness findings across diverse healthcare systems.

### 4.1 Strengths and limitations

This scoping review integrates findings from 11 studies conducted over two decades, across multiple care settings, offering an expanding perspective on the economic landscape of TCM in lung cancer care. In addition, both English and Chinese language studies were included, and the majority used large, real-world insurance databases, enhancing the external validity and generalizability of the findings to broader populations. A notable limitation of this review is the geographic concentration of included studies, most of which originated from Taiwan and mainland China. In Taiwan, NHIRD offers high-quality, population-based data within a single-payer, centralized system that formally reimburses and integrates TCM into routine oncology care. These conditions may not be representative of countries with fragmented, privately funded, or less integrative healthcare models. In contrast, studies from mainland China often rely on regional or hospital-based datasets, which may suffer from inconsistencies in cost reporting, limited follow-up, and variable TCM delivery models due to provincial-level differences in insurance coverage and clinical protocols. Moreover, the lack of nationally standardized guidelines for integrative oncology in China further limits the comparability of findings across regions. As such, the cost-effectiveness outcomes observed in these contexts may have limited transferability to other healthcare systems. Future studies conducted in more varied health system environments, including settings where TCM is less established or less reimbursed, are needed to assess broader applicability. Moreover, it should be noted that the included studies varied in methodology (retrospective cohorts, cross-sectional, RCT), care settings (inpatient vs. outpatient), and outcomes (total cost vs. ICER vs. QALY), which limited direct comparability and meta-analytic synthesis. Furthermore, differences in cost components (e.g., inclusion of indirect costs, currency conversion rates, and price years) pose challenges in drawing consistent conclusions. Moreover, only three studies conducted formal cost-effectiveness or utility analyses. In addition, while some studies (e.g., Tang et al.) attempted to control for confounders using propensity score matching, most retrospective analyses are inherently vulnerable to residual confounding and selection bias, especially regarding patient preference or provider-level factors influencing TCM use. Most studies assessed direct medical costs without considering indirect costs, such as productivity loss or caregiver burden, which are important for understanding the societal economic impact of TCM interventions. A China-based nationwide cross-sectional study estimated that indirect medical costs, stemming from productivity loss and caregiver burden, amounted to approximately US $1,413 per patient since diagnosis, underscoring the substantial proportion of economic burden not captured in direct-cost-only analyses (43). As such, the overall economic burden of TCM interventions for lung cancer may be underestimated. Future research should consider adopting a societal perspective to comprehensively capture both direct and indirect costs associated with TCM-based treatment. And last but not least, although this review included Chinese-language studies, the scope was limited to published peer-reviewed articles, potentially excluding relevant gray literature or non-indexed regional evaluations. Future research should address these gaps by conducting prospective cost-effectiveness analyses, ideally incorporating QALY measurements and patient-reported outcomes. Standardized clinical guidelines for integrating TCM into lung cancer care may help reduce unnecessary spending and optimize treatment protocols. Greater transparency in cost reporting, especially in mainland China, is needed to facilitate comparative evaluations across regions. Finally, the studies included span a 21-year period, during which treatment protocols and cost structures changed substantially. While costs were standardized to 2023 USD, clinical comparability across time remains limited. Moreover, comparator definitions (non-TCM or WM) varied between studies, which may limit the internal validity of cost comparisons.

## 5 Conclusion

This scoping review highlights the complex economic landscape of TCM in the treatment of lung cancer. Given the methodological and contextual heterogeneity among included studies, the findings should be interpreted with caution. Moreover, the applicability of cost-effectiveness results to non-Chinese healthcare settings is limited and warrants careful consideration. Among the included studies, only few employed formal cost-effectiveness or cost-utility analysis frameworks, while the others reported simple cost comparisons without evaluating incremental outcomes. Furthermore, the direction of cost findings varied significantly across studies, with some showing higher total costs in TCM users. As such, the current evidence is insufficient to draw definitive conclusions about the cost-effectiveness of TCM, and highlights the urgent need for rigorous, outcome-linked economic evaluations in this area. While TCM shows potential for enhancing survival and complementing conventional therapies, its economic impact is highly context-dependent. Future research should focus on prospective cost-effectiveness analyses using standardized economic evaluation methods, including QALYs and societal cost perspectives. Clear clinical guidelines and policy support are essential to ensure that TCM is used efficiently and equitably as part of comprehensive lung cancer care. In summary, TCM may offer both clinical and economic value for patients with lung cancer, but its cost-effectiveness depends on treatment duration, care setting, and healthcare system integration. Optimizing the role of TCM in oncology will require evidence-informed policies that balance therapeutic benefit with costs and economic measures.
