# Supplementary material for: Cost analysis and cost-effectiveness of traditional Chinese medicine in lung cancer: a scoping review
Source: Front Public Health. 2025 Nov 3;13:1669751. doi: 10.3389/fpubh.2025.1669751 (PMC12620458; doi:10.3389/fpubh.2025.1669751)
Supplement: Supplementary file 1 [file Supplementary_file_1.docx]

Supplementary Material

# Supplementary Tables

| Table S1- Search terms and their alternatives (in English and Chinese databases) | | |
| --- | --- | --- |
| Cost | **Traditional Chinese Medicine** | **Lung cancer** |
| Cost  Cost Analysis  Cost Measure*  Pricing  Cost Comparison*  Cost Effectiveness Analysis  Cost-Effectiveness Analysis  Cost Benefit Analysis  Cost-Benefit Analysis  Cost Effectiveness Ratio  Cost-Effectiveness Ratio  Cost Utility Analysis  Cost-Utility Analysis  Incremental Cost Effectiveness Ratio  Incremental Cost-Effectiveness Ratio  Economic Evaluation | Traditional Chinese Medicine  Integrative Medicine  Complementary Therap*  Alternative Therap*  Complementary Medicine  Alternative Medicine  Herbal Medicine  Chinese Herbal Drugs  Chinese Plant Extracts  Chinese patent medicine  Acupuncture  Moxibustion  Cupping Treatment  Cupping Therap*  Qigong  Tai Chi  Tai Ji | Lung Neoplasm*  Lung Cancer*  Pulmonary Neoplasm*  Pulmonary Cancer*  Cancer of the Lung  Non-Small Cell Lung Cancer  Non-Small Cell Lung Carcinoma  Small Cell Lung Cancer  Small Cell Lung Carcinoma |
| 成本  成本分析  成本测量  成本指标  根据上下文使用  定价  成本比较  成本效果分析  成本收益分析  成本效果比  成本效用分析  增量成本效果比  经济学评价  经济评价 | 中医药  整合医学  补充治疗  替代治疗  补充医学  替代医学  草药治疗  中草药  中药提取物  中成药  针灸  艾灸  拔罐治疗  拔罐疗法  气功  太极  太极拳 | 肺肿瘤  肺癌  肺部肿瘤  肺部癌症  肺部癌  非小细胞肺癌  非小细胞肺癌瘤  小细胞肺癌  小细胞肺癌瘤 |

| Table S2- Search strategy and search results in each database | | | |
| --- | --- | --- | --- |
| Scopus | #1 | ( TITLE-ABS-KEY ( Cost ) OR TITLE-ABS-KEY ( Cost Analysis ) OR TITLE-ABS-KEY ( Cost Measure* ) OR TITLE-ABS-KEY ( Pricing ) OR TITLE-ABS-KEY ( Cost Comparison* ) OR TITLE-ABS-KEY ( Cost Effectiveness Analysis ) OR TITLE-ABS-KEY ( Cost-Effectiveness Analysis ) OR TITLE-ABS-KEY ( Cost Benefit Analysis ) OR TITLE-ABS-KEY ( Cost-Benefit Analysis ) OR TITLE-ABS-KEY ( Cost Effectiveness Ratio ) OR TITLE-ABS-KEY ( Cost-Effectiveness Ratio ) OR TITLE-ABS-KEY ( Cost Utility Analysis ) OR TITLE-ABS-KEY ( Cost-Utility Analysis ) OR TITLE-ABS-KEY ( Incremental Cost Effectiveness Ratio ) OR TITLE-ABS-KEY ( Incremental Cost-Effectiveness Ratio ) OR TITLE-ABS-KEY ( Economic Evaluation ) ) | [4,207,948 results](https://www-scopus-com.ezproxy.ticaret.edu.tr/results/results.uri?s=%28TITLE-ABS-KEY%28Cost%29+OR+TITLE-ABS-KEY%28Cost+Analysis%29+OR+TITLE-ABS-KEY%28Cost+Measure*%29+OR+TITLE-ABS-KEY%28Pricing%29+OR+TITLE-ABS-KEY%28Cost+Comparison*%29+OR+TITLE-ABS-KEY%28Cost+Effectiveness+Analysis%29+OR+TITLE-ABS-KEY%28Cost-Effectiveness+Analysis%29+OR+TITLE-ABS-KEY%28Cost+Benefit+Analysis%29+OR+TITLE-ABS-KEY%28Cost-Benefit+Analysis%29+OR+TITLE-ABS-KEY%28Cost+Effectiveness+Ratio%29+OR+TITLE-ABS-KEY%28Cost-Effectiveness+Ratio%29+OR+TITLE-ABS-KEY%28Cost+Utility+Analysis%29+OR+TITLE-ABS-KEY%28Cost-Utility+Analysis%29+OR+TITLE-ABS-KEY%28Incremental+Cost+Effectiveness+Ratio%29+OR+TITLE-ABS-KEY%28Incremental+Cost-Effectiveness+Ratio%29+OR+TITLE-ABS-KEY%28Economic+Evaluation%29%29&limit=10&origin=searchhistory&sort=plf-f&src=s&sot=b&sdt=b) |
|  | #2 | ( TITLE-ABS-KEY ( Traditional Chinese Medicine ) OR TITLE-ABS-KEY ( Integrative Medicine ) OR TITLE-ABS-KEY ( Complementary Therap* ) OR TITLE-ABS-KEY ( Alternative Therap* ) OR TITLE-ABS-KEY ( Complementary Medicine ) OR TITLE-ABS-KEY ( Alternative Medicine ) OR TITLE-ABS-KEY ( Herbal Medicine ) OR TITLE-ABS-KEY ( Chinese Herbal Drugs ) OR TITLE-ABS-KEY ( Chinese Plant Extracts ) OR TITLE-ABS-KEY ( Chinese Patent Medicine ) OR TITLE-ABS-KEY ( Acupuncture ) OR TITLE-ABS-KEY ( Moxibustion ) OR TITLE-ABS-KEY ( Cupping Treatment ) OR TITLE-ABS-KEY ( Cupping Therap* ) OR TITLE-ABS-KEY ( Qigong ) OR TITLE-ABS-KEY ( Tai Chi ) OR TITLE-ABS-KEY ( Tai Ji ) ) | [636,192 results](https://www-scopus-com.ezproxy.ticaret.edu.tr/results/results.uri?s=%28TITLE-ABS-KEY%28Traditional+Chinese+Medicine%29+OR+TITLE-ABS-KEY%28Integrative+Medicine%29+OR+TITLE-ABS-KEY%28Complementary+Therap*%29+OR+TITLE-ABS-KEY%28Alternative+Therap*%29+OR+TITLE-ABS-KEY%28Complementary+Medicine%29+OR+TITLE-ABS-KEY%28Alternative+Medicine%29+OR+TITLE-ABS-KEY%28Herbal+Medicine%29+OR+TITLE-ABS-KEY%28Chinese+Herbal+Drugs%29+OR+TITLE-ABS-KEY%28Chinese+Plant+Extracts%29+OR+TITLE-ABS-KEY%28Chinese+Patent+Medicine%29+OR+TITLE-ABS-KEY%28Acupuncture%29+OR+TITLE-ABS-KEY%28Moxibustion%29+OR+TITLE-ABS-KEY%28Cupping+Treatment%29+OR+TITLE-ABS-KEY%28Cupping+Therap*%29+OR+TITLE-ABS-KEY%28Qigong%29+OR+TITLE-ABS-KEY%28Tai+Chi%29+OR+TITLE-ABS-KEY%28Tai+Ji%29%29&limit=10&origin=searchhistory&sort=plf-f&src=s&sot=b&sdt=b&sessionSearchId=16cb78fe78da5aa4523616996420e4c0) |
|  | #3 | ( TITLE-ABS-KEY ( Lung Neoplasm* ) OR TITLE-ABS-KEY ( Lung Cancer* ) OR TITLE-ABS-KEY ( Pulmonary Neoplasm* ) OR TITLE-ABS-KEY ( Pulmonary Cancer* ) OR TITLE-ABS-KEY ( Cancer of the Lung ) OR TITLE-ABS-KEY ( Non-Small Cell Lung Cancer ) OR TITLE-ABS-KEY ( Non-Small Cell Lung Carcinoma ) OR TITLE-ABS-KEY ( Small Cell Lung Cancer ) OR TITLE-ABS-KEY ( Small Cell Lung Carcinoma ) ) | [717,359 results](https://www-scopus-com.ezproxy.ticaret.edu.tr/results/results.uri?s=%28TITLE-ABS-KEY%28Lung+Neoplasm*%29+OR+TITLE-ABS-KEY%28Lung+Cancer*%29+OR+TITLE-ABS-KEY%28Pulmonary+Neoplasm*%29+OR+TITLE-ABS-KEY%28Pulmonary+Cancer*%29+OR+TITLE-ABS-KEY%28Cancer+of+the+Lung%29+OR+TITLE-ABS-KEY%28Non-Small+Cell+Lung+Cancer%29+OR+TITLE-ABS-KEY%28Non-Small+Cell+Lung+Carcinoma%29+OR+TITLE-ABS-KEY%28Small+Cell+Lung+Cancer%29+OR+TITLE-ABS-KEY%28Small+Cell+Lung+Carcinoma%29%29&limit=10&origin=searchhistory&sort=plf-f&src=s&sot=b&sdt=b) |
|  | #4 | #1 AND #2 AND #3 | [935 results](https://www-scopus-com.ezproxy.ticaret.edu.tr/results/results.uri?limit=10&origin=searchhistory&sort=plf-f&src=s&sot=comb&sdt=comb&sessionSearchId=0a2a7a5f3aca6d4a3aa62def77b01a1b) |
| Web of Science | #1 | Cost (Topic) or Cost Analysis (Topic) or Cost Measure* (Topic) or Pricing (Topic) or Cost Comparison* (Topic) or Cost Effectiveness Analysis (Topic) or Cost-Effectiveness Analysis (Topic) or Cost Benefit Analysis (Topic) or Cost-Benefit Analysis (Topic) or Cost Effectiveness Ratio (Topic) or Cost-Effectiveness Ratio (Topic) or Cost Utility Analysis (Topic) or Cost-Utility Analysis (Topic) or Incremental Cost Effectiveness Ratio (Topic) or Incremental Cost-Effectiveness Ratio (Topic) or Economic Evaluation (Topic) | [3,108,875](https://www.webofscience.com/wos/woscc/summary/48eaf4c7-c629-43e3-af45-8febd17ee50f-016d890774/relevance/1) |
|  | #2 | Complementary Therap* (Topic) or Alternative Therap* (Topic) or Complementary Medicine (Topic) or Alternative Medicine (Topic) or Herbal Medicine (Topic) or Chinese Herbal Drugs (Topic) or Chinese Plant Extracts (Topic) or Chinese Patent medicine (Topic) or Acupuncture (Topic) or Moxibustion (Topic) or Cupping Treatment (Topic) or Cupping Therap* (Topic) or Qigong (Topic) or Tai Chi (Topic) or Tai Ji (Topic) | [389,871](https://www.webofscience.com/wos/woscc/summary/20026aff-ca9a-44bc-9948-645335800b96-016d895b20/relevance/1) |
|  | #3 | Lung Neoplasm* (Topic) or Lung Cancer* (Topic) or Pulmonary Neoplasm* (Topic) or Pulmonary Cancer* (Topic) or Cancer of the Lung (Topic) or Non-Small Cell Lung Cancer (Topic) or Non-Small Cell Lung Carcinoma (Topic) or Small Cell Lung Cancer (Topic) or Chinese Plant Extracts (Topic) or Chinese Patent medicine (Topic) or Small Cell Lung Carcinoma (Topic) | [535,768](https://www.webofscience.com/wos/woscc/summary/c4788774-ba08-47df-9c7b-ff68902f950a-016d89933a/relevance/1) |
|  | #4 | #1 AND #2 AND #3 | [724](https://www.webofscience.com/wos/woscc/summary/a4f4d725-a4ff-462a-a9be-a59b78029978-016d89abd1/relevance/1) |
| PubMed | #1 | (((((((((((((((Cost[Title/Abstract]) OR (Cost Analysis[Title/Abstract])) OR (Cost Measure*[Title/Abstract])) OR (Pricing[Title/Abstract])) OR (Cost Comparison*[Title/Abstract])) OR (Cost Effectiveness Analysis[Title/Abstract])) OR (Cost-Effectiveness Analysis[Title/Abstract])) OR (Cost Benefit Analysis[Title/Abstract])) OR (Cost-Benefit Analysis[Title/Abstract])) OR (Cost Effectiveness Ratio[Title/Abstract])) OR (Cost-Effectiveness Ratio[Title/Abstract])) OR (Cost Utility Analysis[Title/Abstract])) OR (Cost-Utility Analysis[Title/Abstract])) OR (Incremental Cost Effectiveness Ratio[Title/Abstract])) OR (Incremental Cost-Effectiveness Ratio[Title/Abstract])) OR (Economic Evaluation[Title/Abstract]) | [662,937](https://pubmed.ncbi.nlm.nih.gov/?term=%28%28%28%28%28%28%28%28%28%28%28%28%28%28%28Cost%5BTitle%2FAbstract%5D%29+OR+%28Cost+Analysis%5BTitle%2FAbstract%5D%29%29+OR+%28Cost+Measure%2A%5BTitle%2FAbstract%5D%29%29+OR+%28Pricing%5BTitle%2FAbstract%5D%29%29+OR+%28Cost+Comparison%2A%5BTitle%2FAbstract%5D%29%29+OR+%28Cost+Effectiveness+Analysis%5BTitle%2FAbstract%5D%29%29+OR+%28Cost-Effectiveness+Analysis%5BTitle%2FAbstract%5D%29%29+OR+%28Cost+Benefit+Analysis%5BTitle%2FAbstract%5D%29%29+OR+%28Cost-Benefit+Analysis%5BTitle%2FAbstract%5D%29%29+OR+%28Cost+Effectiveness+Ratio%5BTitle%2FAbstract%5D%29%29+OR+%28Cost-Effectiveness+Ratio%5BTitle%2FAbstract%5D%29%29+OR+%28Cost+Utility+Analysis%5BTitle%2FAbstract%5D%29%29+OR+%28Cost-Utility+Analysis%5BTitle%2FAbstract%5D%29%29+OR+%28Incremental+Cost+Effectiveness+Ratio%5BTitle%2FAbstract%5D%29%29+OR+%28Incremental+Cost-Effectiveness+Ratio%5BTitle%2FAbstract%5D%29%29+OR+%28Economic+Evaluation%5BTitle%2FAbstract%5D%29&sort=) |
|  | #2 | (((((((((((((((Cost[Title/Abstract]) OR (Cost Analysis[Title/Abstract])) OR (Cost Measure*[Title/Abstract])) OR (Pricing[Title/Abstract])) OR (Cost Comparison*[Title/Abstract])) OR (Cost Effectiveness Analysis[Title/Abstract])) OR (Cost-Effectiveness Analysis[Title/Abstract])) OR (Cost Benefit Analysis[Title/Abstract])) OR (Cost-Benefit Analysis[Title/Abstract])) OR (Cost Effectiveness Ratio[Title/Abstract])) OR (Cost-Effectiveness Ratio[Title/Abstract])) OR (Cost Utility Analysis[Title/Abstract])) OR (Cost-Utility Analysis[Title/Abstract])) OR (Incremental Cost Effectiveness Ratio[Title/Abstract])) OR (Incremental Cost-Effectiveness Ratio[Title/Abstract])) OR (Economic Evaluation[Title/Abstract]) | [662,937](https://pubmed.ncbi.nlm.nih.gov/?term=%28%28%28%28%28%28%28%28%28%28%28%28%28%28%28Cost%5BTitle%2FAbstract%5D%29+OR+%28Cost+Analysis%5BTitle%2FAbstract%5D%29%29+OR+%28Cost+Measure%2A%5BTitle%2FAbstract%5D%29%29+OR+%28Pricing%5BTitle%2FAbstract%5D%29%29+OR+%28Cost+Comparison%2A%5BTitle%2FAbstract%5D%29%29+OR+%28Cost+Effectiveness+Analysis%5BTitle%2FAbstract%5D%29%29+OR+%28Cost-Effectiveness+Analysis%5BTitle%2FAbstract%5D%29%29+OR+%28Cost+Benefit+Analysis%5BTitle%2FAbstract%5D%29%29+OR+%28Cost-Benefit+Analysis%5BTitle%2FAbstract%5D%29%29+OR+%28Cost+Effectiveness+Ratio%5BTitle%2FAbstract%5D%29%29+OR+%28Cost-Effectiveness+Ratio%5BTitle%2FAbstract%5D%29%29+OR+%28Cost+Utility+Analysis%5BTitle%2FAbstract%5D%29%29+OR+%28Cost-Utility+Analysis%5BTitle%2FAbstract%5D%29%29+OR+%28Incremental+Cost+Effectiveness+Ratio%5BTitle%2FAbstract%5D%29%29+OR+%28Incremental+Cost-Effectiveness+Ratio%5BTitle%2FAbstract%5D%29%29+OR+%28Economic+Evaluation%5BTitle%2FAbstract%5D%29&sort=) |
|  | #3 | ((((((((Lung Neoplasm*[Title/Abstract]) OR (Lung Cancer*[Title/Abstract])) OR (Pulmonary Neoplasm*[Title/Abstract])) OR (Pulmonary Cancer*[Title/Abstract])) OR (Cancer of the Lung[Title/Abstract])) OR (Non-Small Cell Lung Cancer[Title/Abstract])) OR (Non-Small Cell Lung Carcinoma[Title/Abstract])) OR (Small Cell Lung Cancer[Title/Abstract])) OR (Small Cell Lung Carcinoma[Title/Abstract]) | [250,823](https://pubmed.ncbi.nlm.nih.gov/?term=%28%28%28%28%28%28%28%28Lung+Neoplasm%2A%5BTitle%2FAbstract%5D%29+OR+%28Lung+Cancer%2A%5BTitle%2FAbstract%5D%29%29+OR+%28Pulmonary+Neoplasm%2A%5BTitle%2FAbstract%5D%29%29+OR+%28Pulmonary+Cancer%2A%5BTitle%2FAbstract%5D%29%29+OR+%28Cancer+of+the+Lung%5BTitle%2FAbstract%5D%29%29+OR+%28Non-Small+Cell+Lung+Cancer%5BTitle%2FAbstract%5D%29%29+OR+%28Non-Small+Cell+Lung+Carcinoma%5BTitle%2FAbstract%5D%29%29+OR+%28Small+Cell+Lung+Cancer%5BTitle%2FAbstract%5D%29%29+OR+%28Small+Cell+Lung+Carcinoma%5BTitle%2FAbstract%5D%29&sort=) |
|  | #4 | #1 AND #2 AND #3 | [29](https://pubmed.ncbi.nlm.nih.gov/?term=longquery0f7c95e5f96ca0c80086&sort=) |
| CNKI |  | (主题/关键词/摘要: 肺癌 OR 肺肿瘤 OR 肺部肿瘤 OR 肺部癌症 OR 肺部癌 OR 非小细胞肺癌 OR 非小细胞肺癌瘤 OR 小细胞肺癌 OR 小细胞肺癌瘤)  AND  (主题/关键词/摘要: 中医药 OR 整合医学 OR 补充治疗 OR 替代治疗 OR 补充医学 OR 替代医学 OR 草药治疗 OR 中草药 OR 中药提取物 OR 中成药 OR 针灸 OR 艾灸 OR 拔罐治疗 OR 拔罐疗法 OR 气功 OR 太极 OR 太极拳)  AND  (主题/关键词/摘要: 成本 OR 成本分析 OR 成本测量 OR 成本指标 OR 定价 OR 成本比较 OR 成本效果分析 OR 成本收益分析 OR 成本效果比 OR 成本效用分析 OR 增量成本效果比 OR 经济学评价 OR 经济评价) | [9](https://oversea.cnki.net/kns/AdvSearch?dbcode=CFLS&crossDbcodes=CJFQ,CDMD,CIPD,CCND,CYFD,CCJD,BDZK,CISD,CJFQ,CDMD,CIPD,CCND,CYFD,CCJD,BDZK,CISD,CJFN) |
| Wanfang |  | (主题/关键词/摘要: 肺癌 OR 肺肿瘤 OR 肺部肿瘤 OR 肺部癌症 OR 肺部癌 OR 非小细胞肺癌 OR 非小细胞肺癌瘤 OR 小细胞肺癌 OR 小细胞肺癌瘤)  AND  (主题/关键词/摘要: 中医药 OR 整合医学 OR 补充治疗 OR 替代治疗 OR 补充医学 OR 替代医学 OR 草药治疗 OR 中草药 OR 中药提取物 OR 中成药 OR 针灸 OR 艾灸 OR 拔罐治疗 OR 拔罐疗法 OR 气功 OR 太极 OR 太极拳)  AND  (主题/关键词/摘要: 成本 OR 成本分析 OR 成本测量 OR 成本指标 OR 定价 OR 成本比较 OR 成本效果分析 OR 成本收益分析 OR 成本效果比 OR 成本效用分析 OR 增量成本效果比 OR 经济学评价 OR 经济评价) | [1](https://s.wanfangdata.com.cn/advanced-search/paper) |

| Table S3- Quality assessment of included studies using JBI Critical Appraisal Tools based on study design | | | | | | | | | | | | | |
| --- | --- | --- | --- | --- | --- | --- | --- | --- | --- | --- | --- | --- | --- |
| Study, year | **Q1** | **Q2** | **Q3** | **Q4** | **Q5** | **Q6** | **Q7** | **Q8** | **Q9** | **Q10** | **Q11** | **Q12** | **Q13** |
| Tang et al. (2024) | Yes | Yes | Yes | Yes | Yes | Yes | Yes | Yes | Yes | Yes | Yes | - | - |
| Nie et al. (2023) | Yes | Yes | Yes | Yes | Yes | Yes | Yes | Yes | - | - | - | - | - |
| Liao et al. (2013) | Yes | Yes | Yes | Yes | Yes | Yes | Yes | Yes | Yes | Yes | Yes | - | - |
| Liu et al. (2021) | Yes | Yes | Yes | Yes | Yes | Yes | Yes | Unclear | Unclear | Unclear | Yes | - | - |
| Liu et al. (2011) | Yes | Yes | Unclear | Yes | Yes | Unclear | Yes | Yes | - | - | - | - | - |
| Zhu et al. (2024) | Yes | Yes | Yes | Yes | Yes | Yes | Yes | Yes | - | - | - | - | - |
| Wu et al. (2015) | Yes | Yes | Yes | Unclear | Yes | Yes | Yes | Yes | Unclear | Unclear | Yes | - | - |
| Bai et al. (2003) | Yes | Unclear | Yes | No | No | No | Yes | Yes | Yes | Yes | Yes | Yes | Yes |
| Zhao et al. (2016) | Yes | Yes | Yes | Yes | Yes | Yes | Yes | Yes | Unclear | Unclear | Yes | - | - |
| He et al. (2009) | Yes | Yes | Yes | No | No | Yes | Yes | Unclear | Unclear | Unclear | Yes | - | - |
| Chang-ming et al. (2018) | Yes | Yes | Yes | Unclear | Yes | Yes | Yes | Yes | Unclear | Unclear | Yes | - | - |

**
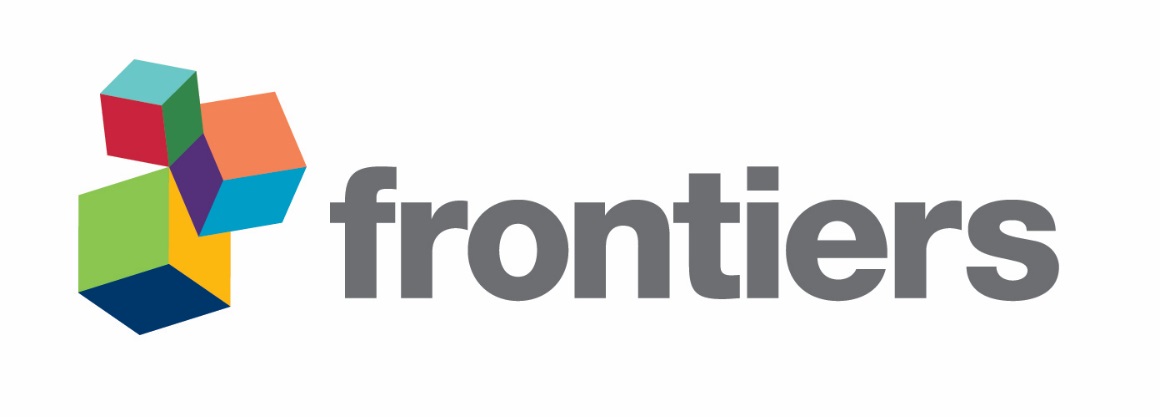
**
